# Wood Decomposition in European Rivers Increases With Temperature but Decreases With Human Population Density

**DOI:** 10.1002/ece3.73821

**Published:** 2026-06-09

**Authors:** Micael Jonsson, Laura Concostrina‐Zubiri, Maria Cristina Bruno, Fernanda Cássio, Giulia Cesarini, Luca Gallitelli, Stefano Larsen, Monika Laux, Giorgio Pace, Cláudia Páscoal, Massimiliano Scalici, Ralf Schulz, José Barquín

**Affiliations:** ^1^ Department of Ecology, Environment and Geoscience Umeå University Umeå Sweden; ^2^ IHCantabria, Instituto de Hidráulica Ambiental Universidad de Cantabria Santander Spain; ^3^ Research and Innovation Centre Fondazione Edmund Mach San Michele all'Adige Italy; ^4^ National Biodiversity Future Center (NBFC) Università di Palermo Palermo Italy; ^5^ Department of Biology, Centre of Molecular and Environmental Biology (CBMA)/Aquatic Research Network (ARNET) Associate Laboratory University of Minho Braga Portugal; ^6^ Institute of Science and Innovation for Bio‐Sustainability (IB‐S) University of Minho Braga Portugal; ^7^ Department of Sciences University of Roma Tre Rome Italy; ^8^ Institute for Environmental Sciences RPTU—University of Kaiserslautern‐Landau Landau Germany

**Keywords:** human population density, land use, latitudinal gradient, plant litter decomposition, riparian zones, streams

## Abstract

Plant litter decomposition in rivers is shaped by multiple environmental conditions, which are modified by riparian zone characteristics and human activities, thereby impacting in situ plant litter decomposition rates. However, disentangling the relative importance of these conditions for plant litter decomposition rates is challenging without large‐scale studies encompassing wide environmental and anthropogenic gradients. We carried out a continental‐scale study on plant litter decomposition in 72 river locations across 7 catchments in Europe (Germany, Italy, Portugal, Spain, Sweden), representing wide gradients in climatic conditions, riparian zone characteristics, land‐use intensity, and human population density. We used remote sensing data and field surveys to quantify catchment, riparian, and river habitat characteristics. To assess decomposition rates, we used standardized wood sticks as a model substrate, representing a globally important source of organic matter in river systems. Wood decomposition rate (percent mass loss per day) increased by 4.3% per 1°C rise in mean annual air temperature but decreased by 3.4% per 10 people increase in population density (per 3.14 km^2^), 2.3% per 100 mm increase in mean annual precipitation, and 0.5% per 1 m increase in channel width. Land‐use intensity and riparian zone characteristics showed no significant effects on wood decomposition rates across the studied gradients. Our results show that wood decomposition rates in the studied rivers are likely to increase linearly with ongoing global warming, reducing the longevity of wood substrates and their reliability as carbon sinks. However, this warming effect might be offset in rivers experiencing concurrent increases in precipitation and human population density. Consequently, the net effect of global change on wood decomposition rates in rivers may be difficult to predict.

## Graphical abstract

We investigated wood decomposition in rivers across seven catchments and 72 river locations in Europe and found that temperature was the main driver of decomposition rate, with a predicted 4.3% increase per 1°C rise in mean annual temperature. However, climate‐change induced increases in mean annual precipitation and an increased human population density could counteract this increase, making the net effect of global change on wood decomposition rates in rivers difficult to predict.

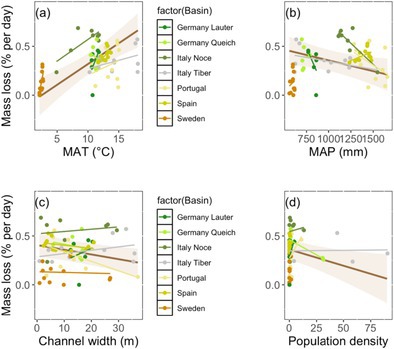

## 1 Introduction

Decomposition of plant litter is essential for nutrient and carbon (C) regeneration in most ecosystems (Polis and Strong 1996; Cebrian 1999) and is receiving increasing attention as a critical driver of ecosystem C balance with implications for the global climate (McClain et al. 2003; Davidson and Janssens 2006; Battin et al. 2009). A range of biotic and abiotic factors are known to control rates of plant litter decomposition; however, which factors exert the strongest influence is still debated (Liu et al. 2024).

In rivers, plant litter decomposition typically proceeds more rapidly than on land, not only because of sufficient moisture, which generally is a limiting factor in terrestrial ecosystems, but also due to physical abrasion caused by the water flow (Gessner et al. 2010; García‐Palacios et al. 2016; Tiegs et al. 2019). The availability and quality of plant litter in rivers are mainly donor controlled, meaning that most of the litter is allochthonous, that is, originating mainly from terrestrial riparian vegetation (Vannote et al. 1980). The input rates and phenology of this organic material, as well as its chemistry and physical structure, are therefore determined by adjacent riparian plant species composition and productivity (Kominoski et al. 2011; Lidman et al. 2017; Pérez et al. 2025). These differences in litter chemistry and physical structure—or quality—among plant species are key regulators of plant litter decomposition in rivers (Ostrofsky 1997; García‐Palacios et al. 2016). In this regard, broadleaf vegetation generally provides faster‐decomposing, higher‐quality litter than does coniferous vegetation (Kominoski et al. 2011; Lidman et al. 2017; Pérez et al. 2025). Also, local light conditions and terrestrial runoff patterns, which are both regulated by riparian zone characteristics, may play an important role, via algal production (Halvorson et al. 2016), water temperature regulation (Johnson 2004), or input of inorganic nutrients (Sponseller and Benfield 2001). The influence of these factors changes naturally along the river continuum, as river width and, thus, canopy openness gradually change from headwaters to downstream reaches (Vannote et al. 1980; Gomi et al. 2002; Buck et al. 2004). Such natural influences may, however, be offset by catchment land uses, especially if anthropic activities occur close to the riparian zone (Feld et al. 2018). Certain types of land use (e.g., agriculture, forestry or urban areas) result in more drastic modifications of riparian zones and can largely increase the input to rivers of inorganic nutrients and novel chemicals, such as pesticides and fungicides, directly impacting the organisms involved in organic matter decomposition (Rasmussen et al. 2012; Fernández et al. 2015). Hence, although elevated nitrate or phosphorus concentrations from runoff can stimulate decomposition via enhanced microbial and invertebrate decomposer activity (Gulis et al. 2006; McKie and Malmqvist 2009; Fernandes et al. 2014; Lima‐Fernandes et al. 2015; Rosemond et al. 2015), eutrophication often introduces co‐occurring stressors, such as oxygen depletion, sedimentation, and contaminants, which negatively impact decomposer communities, thereby offsetting nutrient‐driven enhancements, ultimately reducing decomposition (Pascoal et al. 2005; Rasmussen et al. 2012; Fernández et al. 2015, 2016; Pereira et al. 2016).

Over large spatial scales, climate is a strong driver of organic matter decomposition in rivers. As air temperature increases (IPCC 2023), so does water temperature, especially in smaller streams (Gomi et al. 2002), and high temperatures generally stimulate decomposer activities (e.g., Fernandes et al. 2009; Shah 2021). However, such a temperature effect is not necessarily linear, as exceedingly high temperatures or drought conditions (Simões et al. 2022), can reduce oxygen levels (Bruder et al. 2016), and hamper microbial activity, slowing decomposition. Further, an intact riparian zone may weaken the link between air and water temperatures via shading (Rutherford et al. 1997) or groundwater input (Kaandorp et al. 2019), and plant litter characteristics (e.g., recalcitrance) can modulate the stimulating effects of high water temperature on decomposition (Bruder et al. 2016; Monroy et al. 2023). Nevertheless, while previous large‐scale studies have found that water temperature (Boyero et al. 2011; Follstad Shah et al. 2016) has a strong influence on litter decomposition, few studies have investigated whether large‐scale patterns in air temperature similarly can predict plant litter decomposition in rivers.

Climate change also influences precipitation regimes, altering the intensity and frequency of extreme events, such as spates and droughts. While the effects of drought and flow intermittency on litter decomposition are well‐documented (e.g., Bruder et al. 2011; Simões et al. 2022), the impacts of increased precipitation, elevated discharge, and altered rainfall variability remains poorly understood.

To investigate litter decomposition and its drivers in rivers over large spatial scales, standardized substrates are often used because they remove the influence of large‐scale variation in litter chemistry (i.e., regional differences in riparian plant community composition) on decomposition rates, allowing investigations to focus on other potential biotic and abiotic drivers. Previous large‐scale studies of plant litter decomposition in rivers have used either standardized cotton strips (Tiegs et al. 2007, 2013, 2019) or a standardized wood substrate (Young et al. 2008; Arroita et al. 2012; Estevez et al. 2017). While cotton strips mainly consist of cellulose (Tiegs et al. 2013) and therefore represent an unnatural, albeit standardized, substrate, wood constitutes an important and long‐lived resource for many aquatic organisms in rivers (Wondzell and Bisson 2003), as well as a long‐lasting C stock (Peters‐Collaer et al. 2025) due to its slow decomposition rate compared to other plant litter types (Bilby 2003). Hence, investigations of wood decomposition highlight key processes and functions involved in the decomposition of slowly degradable organic matter in rivers globally.

In this study, we used wooden sticks as a standardized substrate to investigate plant litter decomposition in rivers across broad biogeographical gradients of climate and riparian land‐use intensity (Larsen et al. 2023). We hypothesized that (H1) wood decomposition rates would increase with air temperature, consistent with results of previous studies. However, our main objective was to determine how riparian zone characteristics influence litter decomposition rates in rivers, by examining the effects of riparian plant community composition, riparian canopy openness, and riparian land‐use intensity. Specifically, we hypothesized that wood decomposition rates would (H2) increase with canopy openness, (H3) increase with land‐use intensity, and (H4) be higher in rivers bordered by broadleaf or mixed forests compared to those dominated by coniferous forests.

## 2 Materials and Methods

**FIGURE 1:**
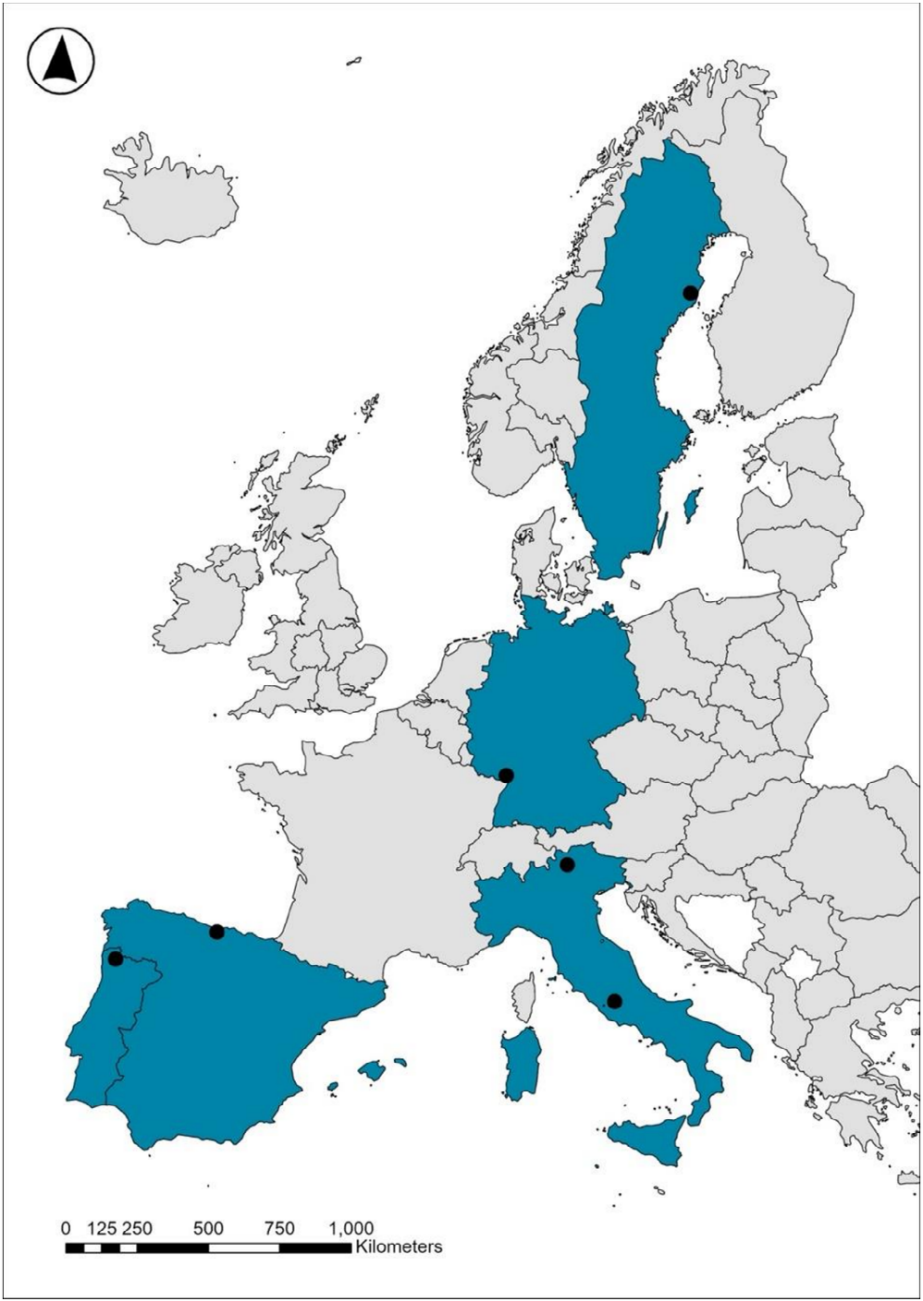
Map showing participating countries (Portugal, Spain, Italy, Germany, and Sweden) in dark blue with each basin marked as a black dot. The German basins Lauter and Queich are represented by one black dot, because of proximity to each other.

RIPARIANET (Larsen et al. 2023) is a Biodiversity+ funded project with five partner countries, viz. Germany, Italy, Portugal, Spain, and Sweden. Portugal, Spain, and Sweden each includes one study basin, whereas Germany and Italy include two (Figure 1, Table 1). For each basin, we used the NetMap software (Benda et al. 2016) to create virtual watersheds and delineate synthetic river networks from digital elevation models (DEM) and historical precipitation data (see Table S1). The virtual watersheds were discretized into relevant landforms, such as reach drainage area and riparian zones (i.e., defined in this study as the inundation polygon produced at 2 bankfull depths; see more details in Fernandez et al. 2012), and synthetic river networks were divided into hydrologically similar river reaches with a maximum length of 1000 m. Each river reach was characterized by three environmental and hydrological variables: elevation, as a proxy of temperature and precipitation; drainage area, as a proxy of discharge, and the percentage cover of forest (i.e., forest cover) in the riparian zone, as a proxy of its conservation status. Mean elevation values were obtained from the DEMs, the drainage area was extracted from the virtual watershed information, and the percentage cover of riparian forest in a 100‐m wide buffer (50 m on each side of river) was calculated using the CORINE Land Cover 2018 map (CLC) from the Copernicus Land Monitoring Service (CLMS). The selection of CLC categories to calculate forest cover was basin specific. In the case of Portugal, Spain, and Germany, the selected categories were ‘Broadleaved forest’ and ‘Mixed forest’, while for Italy and Sweden the ‘Coniferous forest’ category was also included. Then, 12 sampling sites were selected in each basin, except for Germany where 6 sites per basin were selected, based on accessibility and to capture the maximum variation in the elevation, drainage area and riparian forest cover gradients (see Tables S1–S5, for details). In addition, canopy openness was characterized in each site. All selected sites had riparian zones that were to some extent vegetated by trees and/or large shrubs. The selected sites therefore represented a gradient from high (~90%) to low (~10%) canopy openness (for measurement method, see below), with most of the sites (84%) having a forest cover of more than 50%.

**TABLE 1:** Participating countries and their basins, including range of map coordinates, mean elevation (m above sea level), and mean area (km^2^) for each basin. The map coordinates are from the World Geodetic System 1984 (WGS84).

Country	Basin	Map coordinates		Elevation	Catchment area
		*x*	*y*		
Sweden	Sävar River	20.2 to 20.6	63.9–64.4	7–240	194
Germany	Lauter	7.8 to 8.2	49.0–49.2	109–207	231
	Queich	7.9 to 8.3	49.2–49.2	110–197	180
Italy	Noce	10.9 to 11.1	46.2–46.5	215–1178	417
	Tiber	12.5 to 13.3	41.9–42.1	10–818	486
Spain	Pas	−4.2 to −4.3	43.1–43.3	107–430	256
Portugal	Cávado	−7.9 to −8.7	41.6–41.8	18–902	115

### 2.1 Estimation of Wood Decomposition

During the 2024 summer season (from May to June, depending on the climatic region), standardized substrates were deployed at a central location in each sampling site to estimate wood decomposition. The standardized substrate consisted of untreated wooden sticks (15 × 2 × 0.2 cm) made of *Populus × canadensis* (Estevez et al. 2017), which were pre‐weighed to obtain initial mean dry mass (DM). At each site, three sets of five wooden sticks were deployed. The sticks in each set were joined by a nylon string (with plastic separators to avoid sticks from bunching up), which was anchored to a streamside rock or tree with a metal wire. The three sets of wooden sticks were collected after up to ~120 days (i.e., October 2024). At collection, the sticks were gently cleaned from biofilm and stored in cooling boxes, before being brought to the laboratory and frozen at −20°C for later thawing, drying and weighing to estimate mass loss (percent mass lost from initial mass) (Estevez et al. 2017). When the sticks were found broken at collection, mass loss was based on the stick area remaining. Only one set of five sticks (i.e., the best preserved) was used to estimate wood decomposition for each site. The other two sets were spares, but at six sites (four in Noce basin and two in Cávado basin), all sets of sticks were lost due to high discharge. In a few cases, some sticks were found out of water at the time of retrieval, due to temporarily high discharge or reduced water levels (i.e., drought), but because mass loss values of those sticks did not deviate significantly from other values within its basin, it was assumed that this was caused by recent events (i.e., no adjustments of mass loss values were made, and no sticks were excluded from statistical analysis). To account for the different lengths of deployment among sites and basins, mass loss was divided by the number of days between deployment and collection, to obtain a decomposition rate (i.e., percent mass lost per day) that was used as a response variable in later statistical analyses.

### 2.2 Predictor Variables

As predictor variables for wood decomposition rate within and among basins, we selected site‐specific elevation (m a.s.l.), mean annual air temperature (MAT; °C), within‐year variation (CV) in air temperature, mean annual precipitation (MAP; mm), within‐year variation (CV) in precipitation, channel width (m), channel depth (m), canopy openness (%), riparian plant community composition, human population density (number of inhabitants per km^2^), and land‐use intensity. Below, we describe how we calculated these variables.

Elevation was obtained from DEMs, whereas MAT, MAP, and variation in temperature and precipitation were obtained from regional data, where available, or global products for the last 30 years (Table S1). Channel width and depth were extracted from the virtual river networks created with NetMap (see Supporting Information). Mean canopy openness for each site was obtained from hemispheric pictures (*n* = 3). The hemispheric pictures were taken from the centre of the channel (or the closest possible position) with a fish‐eye lens adaptor (ANSTA MPPA007AB) on a mobile phone mounted on a tripod, with the top of the picture facing north. These pictures were processed with the software ImageJ version 1.54 g to calculate the percentage of the image with clear sky as an indicator of canopy openness. Plant community composition was characterized in the riparian zone at the patch level using photointerpretation and ground‐truthing during the 2023 summer season. To evaluate the effect of riparian plant composition on wood decomposition in rivers, we used two composite variables obtained from a principal component analysis (PCA) as predictor variables in the statistical analysis. The primary component (PC1) from the PCA was positively related to cover of coniferous forest and grassland and negatively related to cover of broadleaf forest and shrubland, and the secondary component (PC2) was positively related to mixed forest cover (Figure S1).

Human population density was obtained from GHSL—Global Human Settlement Layer (https://human‐settlement.emergency.copernicus.eu/ghs_pop.php) and was calculated as the number of inhabitants within a 1‐km radius (3.14 km^2^) from the centre of each site. Further, a land‐use index (LUI) was calculated for a 100‐m wide buffer (50 m on each river side) at each site, as an estimate of generic anthropogenic pressure. This calculation consisted of a weighted mean for different types of land use and their percentage of occupation, using a scoring system outlined by Feld (2004):

$$\begin{array}{c} \mathrm{LUI}=5\times \left(\%\text{artificial surface}\right)\\ \;+3\times \left(\%\text{agricultural surface}\right)+\left(\%\text{pasture}\right) \end{array}$$

Hence, the LUI obtained values from 0 (very high naturalness) to 5 (very high anthropogenic impact) (Erba et al. 2015; Pace et al. 2022).

### 2.3 Statistical Analysis

To identify the drivers of wood decomposition rate, we used a linear mixed‐effect model (package ‘lme4’; Bates et al. 2015) with ‘basin’ as a random effect, as sites within a basin are spatially autocorrelated. Elevation (m a.s.l.), MAT (°C), LUI, channel width (m), MAP (mm), CV of both MAT and MAP, channel depth (m), human population density (number per 3.14 km^2^), and riparian plant community composition (i.e., PC1 and PC2; Figure S1) were used as fixed factors. To investigate plausible non‐linear effects and effects of relevant interactions, the quadratic terms (i.e., non‐linear influences) of all fixed factors except for riparian plant community composition, as well as the interaction term MAT × LUI × channel width, were included in the initial model. The three‐way interaction, and its two‐way interactions, were viewed particularly interesting to evaluate statistically, as the impact of land use on rivers may vary with river size, and climate (MAT) may interact with land use to either mitigate or magnify its impact. Model simplification was achieved by backward stepwise removal of non‐significant variables and interactions, starting with quadratic terms and the most complex interaction terms, guided by Akaike Information Criterion (AIC), to obtain the most parsimonious (i.e., best‐fitting) model. As linear mixed‐effects models do not have an exact *F*‐distribution for fixed effects, it is not standard to get *F*‐values and corresponding denominator degrees of freedom in ‘lmer’ models. We therefore used the package ‘lmerTest’ (Kuznetsova et al. 2017), to obtain, and to be able to report, *F*‐values and corresponding numerator and denominator degrees of freedom.

Analysis of variance (ANOVA) and subsequent Tukey's HSD test were used to quantify differences among basins in selected predictor variables as well as in wood decomposition rate. For the ANOVAs, each response variable was checked for normality and was log‐transformed if necessary to meet the requirement of parametric statistical tests. If normality was not achieved, a Krustal–Wallis test was used instead, together with Wilcoxon rank sum test for pairwise comparisons of basins and the Benjamini–Hochberg method to account for multiple comparisons. For the more complex, linear‐mixed effect models, model quality, including variance inflation factor (VIF), was tested using the ‘check_model’ function in the package ‘performance’ (Lüdecke et al. 2021). All statistical analyses were performed using R (R Core Team 2025).

## 3 Results

Climatic variables differed significantly across most basins, whereas differences were small or non‐existent for stream physical and riparian characteristics, human population density, and land‐use intensity (Table 2).

**TABLE 2:** Mean values for predictor variables and wood mass loss for each basin.

	Basin						
	Sweden	Germany Lauter	Germany Queich	Italy Noce	Italy Tiber	Spain	Portugal
Mean annual temperature (°C)	2.4^c^	11.0^b^	11.2^b^	9.0^b^	14.3^a^	12.8^a^	13.4^a^
Temperature variability (CV)	670.9^a^	64.8^c^	64.3^c^	95.1^b^	54.4^d^	36.5^e^	34.2^e^
Mean annual precipitation (mm)	593.9^e^	824.4^c^	721.8^d^	1226.7^b^	999.3^bcd^	1389.2^a^	1488.5^a^
Precipitation variability (CV)	5.0^c^	4.9^c^	4.8^d^	5.7^b^	5.9^b^	5.6^b^	7.4^a^
Channel width (m)	9.6^a^	16.4^a^	14.1^a^	13.7^a^	18.1^a^	9.3^a^	11.1^a^
Channel depth (m)	1.1^a^	1.6^a^	1.5^a^	1.4^a^	1.5^a^	1.2^a^	1.2^a^
Canopy cover (%)	63.2^a^	77.2^a^	69.4^a^	58.3^a^	71.7^a^	63.7^a^	59.0^a^
Human population (3.14 km^−2^)	0.3^b^	1.2^ab^	10.8^ab^	2.3^a^	17.8^ab^	0.4^b^	2.0^a^
Land use intensity (LUI)	0.9^a^	1.1^a^	1.5^a^	0.9^a^	1.9^a^	0.7^a^	1.7^a^
Wood mass loss (% day^−1^)	0.12^c^	0.33^b^	0.39^b^	0.55^a^	0.35^b^	0.41^ab^	0.31^b^

*Note:* Small, lower‐case letters indicate significant pairwise differences between basins.

**FIGURE 2:**
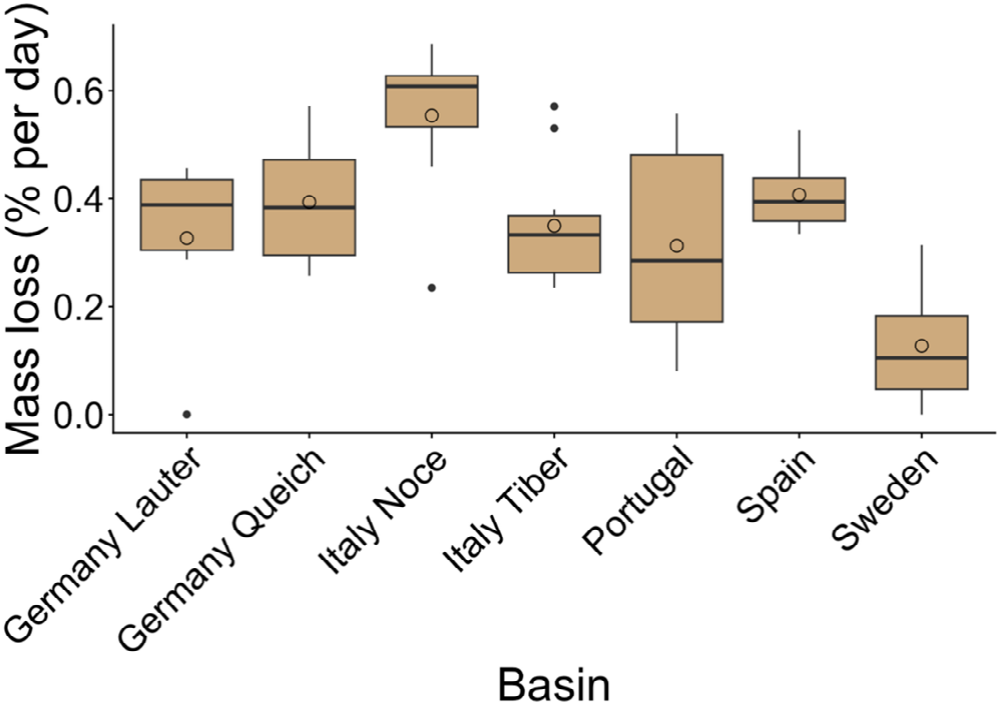
Boxplot of wood decomposition rate for seven basins across Europe. The boxplots show the minimum and maximum excluding outliers, first quartile (median of the lower half), median, and third quartile (median of upper half). Open circles represent the mean value for each basin.

Wood decomposition rate was significantly higher for Italy Noce and lower for Sweden than for the other basins (*F* = 9.890 *p* < 0.001; Table 2, Figure 2).

**FIGURE 3:**
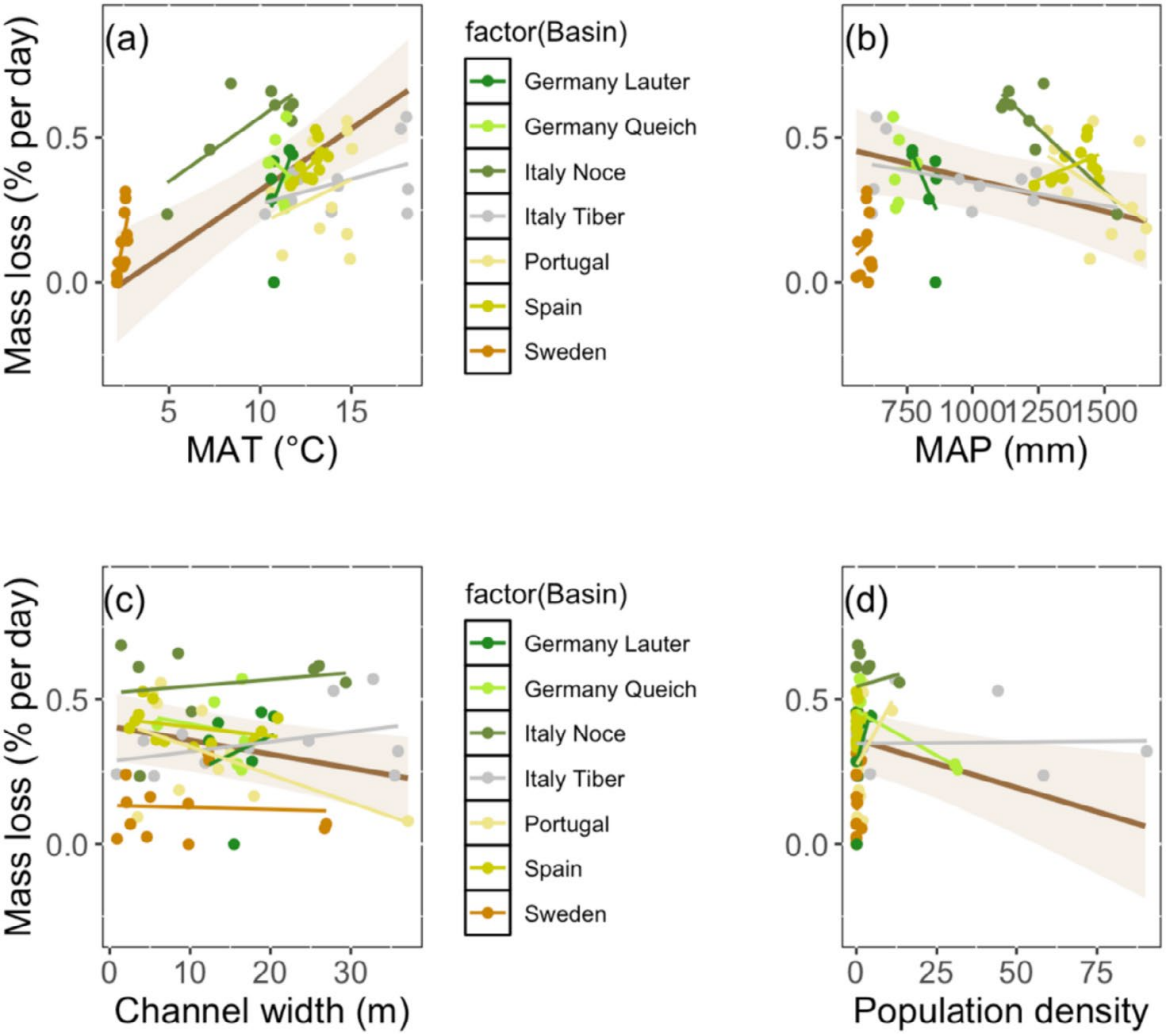
Marginal effects (brown line) showing the predicted wood decomposition rate across gradients in (a) mean annual temperature (MAT), (b) mean annual precipitation (MAP), (c) channel width, and (d) human population density (3.14 km^−2^), from the best‐fitting linear mixed‐effect model. Light brown color represents 95% confidence band. Data points show raw data, with a linear regression fitted for each basin.

The best‐fitting model for wood decomposition rate included a linearly positive influence of MAT (*F*_1,21.4_ = 20.66, *p* < 0.001; Figure 3a), a linearly negative influence of MAP (*F*_1,24.5_ = 4.86, *p* = 0.037; Figure 3b), a linearly negative influence of channel width (*F*_1,56.5_ = 6.34, *p* = 0.015; Figure 3c), and a linearly negative influence of human population density (*F*_1,56.3_ = 6.42, *p* = 0.014; Figure 3d). VIFs for predictors included in the best‐fitting model were very low (< 2), and no non‐linear influences were detected. Model marginal *R* ^2^ was 0.366, while conditional *R* ^2^ (i.e., including the random effect) was 0.758, indicating an important contribution from among‐basin differences. Based on partial regression results on predicted wood decomposition rate (Figure 3a–d), there was a ~4.3% increase in decomposition rate with every 1°C increase, a ~2.3% decrease in decomposition rate with every 100 mm increase in precipitations, a ~0.5% decrease in decomposition rate with every 1 m increase in channel width, and a ~3.4% decrease in decomposition rate with every 10 people increase in population density (Figure 3).

## 4 Discussion

We found strong effects of climatic conditions—that is, mean annual temperature and precipitation—on wood decomposition rate in rivers across Europe. As we hypothesized (H1), air temperature had a strong positive effect on wood decomposition rates, consistent with previous large‐scale studies showing water temperature as a key driver of organic matter decomposition in streams and rivers (Boyero et al. 2011; Follstad Shah et al. 2016). In contrast, we found no support for the hypothesized effects of riparian canopy openness, land‐use intensity, or riparian tree species composition on wood decomposition rates (H2–H4), which can be partly explained by the relative intactness of the riparian zones in our study reaches. In other words, despite the gradient in canopy openness, we lacked sites with no canopy cover. Nevertheless, the observed negative relationship between human population density and decomposition rates indicates that human activities still influence organic matter turnover in rivers (Woodward et al. 2012; Tiegs et al. 2024), though this pattern was mostly driven by the high population density in the Tiber basin.

Although hypotheses H2–H4 were not supported, our results reinforce the dominant role of climate, particularly temperature, in regulating organic matter decomposition in rivers at broad spatial scales. For example, Follstad Shah et al. (2016) found a 5%–21% increase in litter loss associated with a 1°C–4°C rise in water temperature. Our estimated ~4% increase in wood decomposition rate per 1°C increase in air temperature falls at the lower end of that range, which is expected given that wood is more recalcitrant and therefore decomposes more slowly compared to leaf litter (Gulis et al. 2004; Peters‐Collaer et al. 2025). Interestingly, our results indicate that air temperature is a good predictor of wood decomposition rates in rivers, despite most of our study sites (84%) having a substantial canopy cover (51%–89%), which should provide shading and thus buffer water temperatures from high air temperatures (Johnson 2004). Indeed, previous modeling and empirical studies indicate a general increase in stream temperature in parallel with increasing air temperature (e.g., van Vliet et al. 2013; Grey et al. 2023). Accordingly, although we did not directly measure water temperature at our sites, our results suggests that, at continental scales, air temperatures override local thermal buffering, such as from shading (Rutherford et al. 1997) or groundwater input (Kaandorp et al. 2019); this raises the question about the extent to which riparian cover can mitigate air warming effects on river ecosystem processes.

The observed negative effect of precipitation on wood decomposition rate was unexpected, considering that moisture generally promotes decomposition. Moreover, higher precipitation typically leads to greater runoff and discharge, which can increase physical abrasion, causing mechanical mass loss, and nutrient inputs and enhanced oxygen availability, which should stimulate microbially mediated decomposition (e.g., Bruder et al. 2016; Sponseller and Benfield 2001). However, we found that higher overall precipitation instead was associated with reduced wood decomposition rates. Further, the lack of significant effect of precipitation variance suggests that extreme hydrological events (i.e., drought and spates) were of low importance for wood decomposition rate compared to annual precipitation levels. While we find the negative effect of precipitation on wood decomposition rates unexpected, a possible explanation could be that higher water volumes dilute microbial communities or the dissolved nutrients required for microbial decomposition, thereby limiting their ability to degrade recalcitrant substrates, such as wood. Another, non‐mutually exclusive explanation could be that greater precipitation reduces water temperatures, potentially constraining microbial activity. However, as the effect of (air) temperature was included in the statistical model, this latter mechanism is unlikely to explain the observed pattern.

A potential concern is whether our dataset had sufficient variation to disentangle the effects of climate, environmental conditions, and land‐use intensity on wood decomposition rates in rivers. As expected, mean annual temperature and precipitation showed strong gradients and significant differences among basins (Figure S2, Table 2), reflecting their large spatial scale. In contrast, variables such as land‐use intensity were expected to vary more within than among basins, as human population density tends to increase from headwaters to the main stem within river networks (Fang and Jawitz 2019). Furthermore, as canopy cover to a large degree is a function of river size (i.e., channel width), this would also be expected to vary more within basins than among basins, consistent with our study design. Our results mostly support these expectations, as channel width, land‐use intensity, and canopy cover differed substantially within river basins but not among them (Figure S3, Table 2). Interestingly, however, local human population density differed among basins, indicating that direct human presence was not the major driver of differences in land‐use intensity or canopy openness. This may reflect the spatial segregation of land uses; agricultural impacts are typically concentrated in rural areas, whereas high population densities characterize urban centres (Rounsevell et al. 2006). This underscores that the anthropogenic pressures exerted on rivers and riparian zones are highly dependent on the specific type of land use. Nevertheless, the observed large‐scale variation in population density in our study is primarily due to the extensive north‐to‐south gradient, encompassing highly populated urban areas in the south to largely unpopulated areas in the north, which therefore overrides the expected, and in most basins observed, increase in human population density with increasing river size. Overall, our study design captured a large variation in key predictors, although these variables operated at different spatial scales.

Taken together, our results indicate that wood decomposition rates in rivers are likely to increase with climate warming, largely independent of land‐use intensity. Over the long term, this may decrease the C‐storage capacity of rivers and diminish the persistence of woody debris as a contributor to river habitat complexity and as a food resource to decomposer organisms. However, our findings also suggest that other co‐occurring environmental changes may exert counteracting effects. For instance, warming‐induced increases in decomposition rates may be offset by increased levels of precipitation or increased human population density. Consequently, the net effect of global change on wood decomposition rates in rivers may be difficult to predict.

## Author Contributions

**Micael Jonsson:** conceptualization (equal), data curation (equal), formal analysis (lead), funding acquisition (equal), investigation (equal), methodology (equal), project administration (equal), resources (equal), software (equal), supervision (equal), validation (equal), visualization (equal), writing – original draft (lead), writing – review and editing (lead). **Laura Concostrina‐Zubiri:** conceptualization (equal), data curation (lead), formal analysis (lead), funding acquisition (equal), investigation (equal), methodology (lead), project administration (equal), resources (equal), software (equal), supervision (equal), validation (equal), visualization (equal), writing – original draft (equal), writing – review and editing (equal). **Maria Cristina Bruno:** conceptualization (equal), data curation (equal), formal analysis (equal), funding acquisition (equal), investigation (equal), methodology (equal), project administration (equal), resources (equal), software (equal), supervision (equal), validation (equal), visualization (equal), writing – original draft (equal), writing – review and editing (equal). **Fernanda Cássio:** conceptualization (equal), data curation (equal), formal analysis (equal), funding acquisition (equal), investigation (equal), methodology (equal), project administration (equal), resources (equal), software (equal), supervision (equal), validation (equal), visualization (equal), writing – original draft (equal), writing – review and editing (equal). **Giulia Cesarini:** conceptualization (equal), data curation (equal), formal analysis (equal), funding acquisition (equal), investigation (equal), methodology (equal), project administration (equal), resources (equal), software (equal), supervision (equal), validation (equal), visualization (equal), writing – original draft (equal), writing – review and editing (equal). **Luca Gallitelli:** conceptualization (equal), data curation (equal), formal analysis (equal), funding acquisition (equal), investigation (equal), methodology (equal), project administration (equal), resources (equal), software (equal), supervision (equal), validation (equal), visualization (equal), writing – original draft (equal), writing – review and editing (equal). **Stefano Larsen:** conceptualization (equal), data curation (equal), formal analysis (equal), funding acquisition (equal), investigation (equal), methodology (equal), project administration (lead), resources (equal), software (equal), supervision (equal), validation (equal), visualization (equal), writing – original draft (equal), writing – review and editing (equal). **Monika Laux:** conceptualization (equal), data curation (equal), formal analysis (equal), funding acquisition (equal), investigation (equal), methodology (equal), project administration (equal), resources (equal), software (equal), supervision (equal), validation (equal), visualization (equal), writing – original draft (equal), writing – review and editing (equal). **Giorgio Pace:** conceptualization (equal), data curation (equal), formal analysis (equal), funding acquisition (equal), investigation (equal), methodology (equal), project administration (equal), resources (equal), software (equal), supervision (equal), validation (equal), visualization (equal), writing – original draft (equal), writing – review and editing (equal). **Cláudia Páscoal:** conceptualization (equal), data curation (equal), formal analysis (equal), funding acquisition (equal), investigation (equal), methodology (equal), project administration (equal), resources (equal), software (equal), supervision (equal), validation (equal), visualization (equal), writing – original draft (equal), writing – review and editing (equal). **Massimiliano Scalici:** conceptualization (equal), data curation (equal), formal analysis (equal), funding acquisition (equal), investigation (equal), methodology (equal), project administration (equal), resources (equal), software (equal), supervision (equal), validation (equal), visualization (equal), writing – original draft (equal), writing – review and editing (equal). **Ralf Schulz:** conceptualization (equal), data curation (equal), formal analysis (equal), funding acquisition (equal), investigation (equal), methodology (equal), project administration (lead), resources (equal), software (equal), supervision (equal), validation (equal), visualization (equal), writing – original draft (equal), writing – review and editing (equal). **José Barquín:** conceptualization (equal), data curation (lead), formal analysis (lead), funding acquisition (equal), investigation (equal), methodology (lead), project administration (equal), resources (equal), software (equal), supervision (equal), validation (equal), visualization (equal), writing – original draft (equal), writing – review and editing (equal).

## Funding

This work was supported by Fundação para a Ciência e a Tecnologia (FCT) (LA/P/0069/2020), Contrato‐Programa (UID/04050/2025), Fundação para a Ciência e a Tecnologia (FCT), Swedish Research Council (2022‐01745), European Commission (101052342), Ministero dell'Università e della Ricerca (MUR), MICIU/AEI/10.13039/501100011033/the European Union NextGenerationEU/PRTR, MICIU/AEI/10.13039/501100011033 (RYC2023‐042640‐I), and Deutsche Forschungsgemeinschaft (SCHU2271/22‐1).

## Conflicts of Interest

The authors declare no conflicts of interest.

## Supporting information


**Figure S1:** Results from a PCA on riparian plant community composition (relative cover) across all sampling sites (black points).
**Figure S2:** Boxplots of (a) mean annual temperature (MAT), (b) temperature variability (CV), (c) mean annual precipitation (MAP), and (d) variability (CV) in precipitation in seven basins across Europe.
**Figure S3:** Boxplots of (a) channel width, (b) canopy cover, (c) human population density, and (d) land‐use intensity in seven basins across Europe.
**Table S1:** Summary of DEM and precipitation data used to delineate the synthetic river networks.
**Table S2:** Variable range for the spatial and environmental gradients.
**Table S3:** Summary of the predictor variables.
**Table S4:** Summary of environmental predictor data sources and details.
**Table S5:** Identification of the green‐up peak period based on the Copernicus product parameter “Season maximum (date)” for each basin.

## Data Availability

The data supporting the results of this study are openly available on Figshare at https://doi.org/10.6084/m9.figshare.31572271.
