# Supplementary material for: Wood Decomposition in European Rivers Increases With Temperature but Decreases With Human Population Density
Source: Ecol Evol. 2026 Jun 9;16(6):e73821. doi: 10.1002/ece3.73821 (PMC13247557; doi:10.1002/ece3.73821)
Supplement: Supplementary file 1 — Figure S1: Results from a PCA on riparian plant community composition (relative cover) across all sampling sites (black points). Figure S2: Boxplots of (a) mean annual temperature (MAT), (b) temperature variability (CV), (c) mean annual precipitation (MAP), and (d) variability (CV) in precipitation in seven basins across Europe. Figure S3: Boxplots of (a) channel width, (b) canopy cover, (c) human population density, and (d) land‐use intensity in seven basins across Europe. Table S1: Summary of DEM and precipitation data used to delineate the synthetic river networks. Table S2: Variable range for the spatial and environmental gradients. Table S3: Summary of the predictor variables. Table S4: Summary of environmental predictor data sources and details. Table S5: Identification of the green‐up peak period based on the Copernicus product parameter “Season maximum (date)” for each basin. [file ECE3-16-e73821-s001.docx]

**SUPPORTING INFORMATION FOR:**

**WOOD DECOMPOSITION IN EUROPEAN RIVERS INCREASES WITH TEMPERATURE BUT DECREASES WITH HUMAN POPULATION DENSITY**

Micael Jonsson^1*^, Laura Concostrina-Zubiri^2^, Maria Cristina Bruno^3,4^, Fernanda Cássio^5,6^, Giulia Cesarini^7^, Luca Gallitelli^7^, Stefano Larsen^3,4^, Monika Laux^8^, Giorgio Pace^5,6^, Cláudia Páscoal^5,6^, Massimiliano Scalici^7^, Ralf Schulz^8^, José Barquín^2^

^1^Department of Ecology, Environment and Geoscience, Umeå University, 90187 Umeå, Sweden

^2^IHCantabria, Instituto de Hidráulica Ambiental, Universidad de Cantabria, Santander, Spain

^3^Research and Innovation Centre, Fondazione Edmund Mach, Via Mach 1, 38098 S. Michele all’Adige, Italy

^4^National Biodiversity Future Center (NBFC), Università di Palermo, Piazza Marina 61, 90133 Palermo, Italy

^5^Centre of Molecular and Environmental Biology (CBMA) / Aquatic Research Network (ARNET) Associate Laboratory, Department of Biology, University of Minho, Braga, Portugal

^6^Institute of Science and Innovation for Bio-Sustainability (IB-S), University of Minho, Braga, Portugal

^7^University of Roma Tre, Department of Sciences, Viale Guglielmo Marconi, 446 00146 Rome, Italy

^8^Institute for Environmental Sciences, RPTU – University of Kaiserslautern-Landau, Landau, Germany

*Corresponding author

E-mail address: micael.jonsson@umu.se

**Supporting Materials and Methods**

**1. Delineation of synthetic river networks**

The delineation of the synthetic river networks was performed following the method described by Benda *et al.* (2011). For each basin, the synthetic river network was generated by inferring flow directions from a high-resolution Digital Elevation Model (DEM) and precipitation data (Table S1), following the algorithms described by Clarke et al. (2008). The resulting river network was divided into segments with a maximum length of 1000 m, which represent the analytical unit used in this study. Then, we used the NetMap software (Benda et al., 2007) to delineate the riparian zones applying a geomorphological approach in which the valley floor elevation is defined as a multiple of the bank full depth (BFD) of each river reach (Fernández et al., 2012). In this study, we adopted a multiplier of 2 BFDs because it usually comprises the active floodplain and the first alluvial plain (Rosgen, 1996) and corresponds to flood levels associated with an approximate 50‑year return interval (Ilhardt et al., 2000). The bankfull depth estimates for every segment of the river network were derived from a regional regression model that relates drainage area and mean annual precipitation to field-derived measurements of bankfull channel depth (see Benda et al., 2011 for further methodological details).

**TABLE S1.** Summary of DEM and precipitation data used to delineate the synthetic river networks.

|  | SAJA | NOCE | ANIENE | CAVADO | LAUTER/ QUEICH | SAVAR |
| --- | --- | --- | --- | --- | --- | --- |
| DEM | 5 m, PNOA | 10 m, Portale Geocartografico Trentino | 10 m, Instituto Nazionale di Geofisica e Vulcanologia (INGV) | 10 m | 5 m, Landesamt für Vermessung und Geobasisinformation Rheinland-Pfalz | 1 m, Lantmäteriet |
| PRECIPI-TATION | WordClim (1970-2000) | WordClim (1970-2000) | WordClim (1970-2000) | WordClim (1970-2000) | WordClim (1970-2000) | WordClim (1970-2000) |

**2. Environmental characterization of river reaches**

To select sampling sites that spanned the spatial and environmental gradients of each case study, river reaches were classified according to three factors: (a) catchment area, (b) elevation, and (c) riparian forest conservation status (Table S2). We defined multiple classes for each factor to capture all combinations of gradients, applying a 50‑m buffer so that, in headwater areas, the selected area included not only the potential riparian zone but also a slightly broader section. Catchment area classes were defined using the total contributing drainage area of each reach. Four classes were established for all case studies except the Queich catchment (DE), which included only three. Elevation classes were limited to two categories in the Savar and Queich basins, and elevation ranges for each basin were adjusted according to local vegetation distribution limits. Riparian forest classes were defined on a case‑specific basis, reflecting the characteristics of each river basin.

**TABLE S2.** Variable range for the spatial and environmental gradients.

|  | CATCHMENT AREA (km^2^) | ELEVATION (m) | FOREST (%) |
| --- | --- | --- | --- |
| Class 1 | > 500 km2 | < 200 m | < 40 % |
| Class 2 | 100 km2 < x < 500 km2 | 200 m < x < 700 m | 40 % < x < 70 % |
| Class 3 | 10 km2 < x < 100 km2 | > 700 m | > 70 % |
| Class 4 | < 10km2 | - | - |

**3. Calculation of environmental predictors**

We obtained the environmental variables for each basin at the reach scale using climate, topographic, soil, and LiDAR data (Table S3). For each reach, average values were computed as the mean of all pixels located within the riparian zone corresponding to that river reach.

**TABLE S3.** Summary of the predictor variables.

|  | VARIABLES | UNITS |
| --- | --- | --- |
| CLIMATE | Mean temperature: annual | ⁰C |
|  | Coefficient of variation: annual mean temperature | Dimensionless |
|  | Maximum temperature: spring | ⁰C |
|  | Maximum temperature: summer | ⁰C |
|  | Maximum temperature: winter | ⁰C |
|  | Minimum temperature: spring | ⁰C |
|  | Minimum temperature: summer | ⁰C |
|  | Minimum temperature: winter | ⁰C |
|  | Mean precipitation: annual | mm |
|  | Coefficient of variation: annual mean precipitation | Dimensionless |
|  | Mean precipitation: spring | mm |
|  | Mean precipitation: summer | mm |
|  | Mean precipitation: winter | mm |
| TOPOGRAPHY | Digital Elevation Model | m |
|  | Slope | Degrees |
|  | Aspect | Degrees |
|  | Solar Radiation | WH/m2 |
|  | Eastness | Dimensionless |
|  | Southness | Dimensionless |
|  | Topographic Wetness Index | Dimensionless |
|  | Euclidean Distance | m |
| SOIL PROPERTIES | Clay content | % |
|  | Silt content | % |
|  | Sand content | % |
|  | Coarse fragments | % |
|  | Bulk density | T/m^3^ |
|  | USDA soil textural classes | - |
|  | Available Water Capacity | Dimensionless |
|  | Lithology | - |
| LIDAR | Average vegetation height | m |
|  | Average vegetation height, 3x3 filter | m |

Whenever possible, data sources corresponded to regional databases or to products with the highest available spatial and temporal resolution (Table S4). All variables were rescaled to 10 m to ensure consistency in spatial data processing. Bilinear interpolation was applied, when necessary, as it is the most suitable method for continuous variables, computing each pixel value as the distance‑weighted average of the four nearest neighbouring pixels.

**TABLE S4.** Summary of environmental predictor data sources and details.

|  | |  | |  | SAJA | | | NOCE | | ANIENE | | CAVADO | | QUEICH/ LAUTER | | SAVAR | |
| --- | --- | --- | --- | --- | --- | --- | --- | --- | --- | --- | --- | --- | --- | --- | --- | --- | --- |
| CLIMATE | |  | | SOURCE | | | Predictia (IHCantabria, 2015) | HIGHLANDER PROJECT  (Raffa *et al.*, 2021) | | HIGHLANDER PROJECT  (Raffa *et al.*, 2021) | | EMO1  (Gomes *et al.*, 2020) | | EMO1  (Gomes *et al.*, 2020) | | SMHI  (Andersson *et al.*, 2021) | |
|  |  |  | | TIME PERIOD | | |  | 1991-2020 | | 1991-2020 | | 1993-2022 | | 1993-2022 | | 1989-2018 | |
| SOIL PROPERTIES | |  | | SOURCE | | | LUCAS topsoil data (Ballabio *et al.*, 2016) | | | | | | | | | | |
| GEOLOGY | |  | | SOURCE | | | Continental Europe surface lithology based on EGDI (Hengl, 2021) | | | | | | | | | | |
| LiDAR | |  | | SOURCE | | | PNOA (CNIG) | Ufficio Sistemi Informativi - Servizio autorizzazioni e valutazioni ambientali | | ETH (Lang *et al.*, 2023) | | ETH (Lang *et al.*, 2023) | | GeoPortal Rhineland-Palatinate - Serviceportal Rheinland-Pfalz & Institut national de l'information géographique et forestière | | Ythöjdmodell Nedladdning | |
|  |  |  | | DATE | | | 2018 | 2014 | | 2020 | | 2020 | | 2022 | | 2019 | |

**3.1 Topographic variables**

Topographic variables were obtained from the DEMs of each study basin using the tools available in geographic information systems (GIS). When necessary, DEMs were resampled to 10 m for the calculation of these variables, applying a bilinear interpolation.

**3.2 Multispectral imagery**

Following the image‑selection methodology described in Álvarez‑Martínez et al. (2018), the selected Sentinel-2 scenes were those with the highest sun elevation, lowest cloud cover, and dates closest to peak greenness:

• Sävarån basin (Sweden): Sentinel‑2 T34VDR and T34WDS

• Cávado basin (Northern Portugal): Sentinel‑2 T29TNF and T29TNG

• Noce basin (Northern Italy): Sentinel‑2 T32TPS

• Aniene basin (Central Italy): Sentinel‑2 T33TTG/T33TQM and T33TUG

• Queich basin (Germany): Sentinel‑2 T32UMV

• Lauter basin (Germany): Sentinel‑2 T32ULV and T32UMV

As proposed in Álvarez‑Martínez et al. (2018), this study followed a three‑year temporal window. The choice of years balanced the benefit of enlarging the dataset against the risk that older imagery might exhibit increased reflectance variability due to habitat succession or other environmental changes over time.

Because the case studies span a wide range of latitudes and climatic conditions, the timing of peak greenness differs among basins. For this reason, the optimal dates for each scene were evaluated individually. This evaluation involved analysing image quality in terms of cloud cover, identifying peak‑greenness timing for each basin, and determining whether snow cover occurred during parts of the year.

To assess Sentinel‑2 image availability based on cloud cover, the Copernicus Browser platform was used. For each basin, monthly image‑availability levels were calculated from the number of scenes with low cloud cover, and these values were then averaged across the three selected years (2021, 2022, and 2023).

To determine peak greenness, we used the Vegetation and Productivity parameters from the Copernicus programme, available at 10‑m spatial resolution. These datasets were downloaded from the WEkEO platform, a free service providing access to Sentinel mission products. The parameters are derived from a Plant Phenology Index (PPI) time series, computed for up to two growing seasons. According to the WEkEO documentation, the PPI is a physically based vegetation index optimised for monitoring vegetation phenology and shows a linear relationship with green leaf area index (LAI).

The Vegetation and Productivity dataset includes parameters such as the start, end, and duration of the growing season; dates of minimum and maximum vegetation index values; the maximum value and its amplitude (difference between maximum and minimum); the slopes during green‑up and green‑down phases; and seasonal and total productivity.

For this study, we focused on the “Season maximum (date)” parameter, defined as the day of the year on which the PPI reaches its maximum, representing the green‑up peak for each pixel. This variable is expressed as a numerical day‑of‑year index, with January 1 as day 1, July 1 as day 182, and December 31 as day 365.

Before analysis, all datasets were clipped to each basin to ensure that only relevant pixels were included. To standardize the information across riverbank pixels, the distribution of PPI maximum‑date values—which showed a clearly Gaussian pattern—was used. The mean and standard deviation were calculated, and from these statistics, a minimum and maximum date were derived representing the period during which most pixels reached their peak greenness or productivity.

Data for this analysis were available for 2021 and 2022 (Table S5), as the computation of these parameters requires a complete annual PPI time series.

**TABLE S5.** Identification of the green-up peak period based on the Copernicus product parameter "Season maximum (date)" for each basin.

| Country | Year | Mean | St Dev | Min date | Max date |
| --- | --- | --- | --- | --- | --- |
| Sweden | 2021 | 187 | 22 | June 14th | July 28th |
| Sweden | 2022 | 195 | 22 | June 22nd | August 5th |
| Portugal | 2021 | 153 | 40 | April 23rd | July 12th |
| Portugal | 2022 | 152 | 38 | April 24th | July 9th |
| Italy (Noce) | 2021 | 181 | 24 | June 6th | July 24th |
| Italy (Noce) | 2022 | 173 | 23 | May 30th | July 15th |
| Italy (Aniene) | 2021 | 155 | 30 | May 5th | July 4th |
| Italy (Aniene) | 2022 | 150 | 29 | May 1st | June 28th |
| Germany (Queich) | 2021 | 179 | 26 | June 2nd | July 24th |
| Germany (Queich) | 2022 | 164 | 26 | May 18th | July 9th |
| Germany (Lauter) | 2021 | 177 | 23 | June 3rd | July 19th |
| Germany (Lauter) | 2022 | 166 | 23 | May 23rd | July 8th |

Finally, periods of snow cover in each basin were also considered, as snow can alter the values of the spectral indices used in this study. For this analysis, Sentinel‑2 imagery was examined for each case study, using the same temporal window applied in the cloud‑cover assessment (April to September). Snow cover was detected in only two basins: the Sävarån basin (Sweden) and the Noce basin (Italy).

Based on all these datasets, a prioritisation scheme was established to determine the months ultimately selected. Primary weight was given to the timing of the PPI maximum, followed by cloud‑cover levels, and lastly snow cover. Although snow cover can be a decisive factor for excluding specific months, in the two basins where it occurred, the months identified as optimal were not affected by the presence of snow.

The cloud mask applied in this study was generated using the following expression in SNAP for each selected scene:

$$if (scl\_cloud\_medium\_proba + scl\_cloud\_high\_proba + scl\_thin\_cirrus)<255$$

$$then 0 else 1$$

After generating the cloud mask, a subset and resampling procedure was applied to this layer together with all relevant spectral bands (B2, B3, B4, B8, B8A, B11, and B12). Bands 8A, 11, and 12 were resampled to a 10‑m spatial resolution to match the resolution of the remaining bands and to ensure consistency across all datasets used in this study.

**3.3 LiDAR for vegetation characterization**

Two variables were derived from the LiDAR data: (i) the average vegetation height, computed using the 99th percentile of maximum vegetation height, and (ii) the average vegetation height after applying a 3×3‑pixel neighbourhood filter.

These variables were extracted, whenever possible, from the Digital Surface Model (DSM). In those study areas where no DSM was available, but native LiDAR point‑cloud files existed, the DSM was generated from the raw data using the LAStools processing toolbox (Isenburg et al., 2008), a collection of scripts that can be integrated into various GIS software environments for LiDAR data processing.

In cases where neither a DSM nor raw LiDAR data were available—specifically, the Cávado catchment (Portugal) and the Aniene catchment (Italy)—we used the high‑resolution canopy height model developed by Lang et al. (2023). This model provides a 10‑m resolution canopy height map for the year 2020, derived from GEDI data (cited), Sentinel‑2 imagery, and convolutional neural networks (CNNs).

**4. Statistical methods**

Each site’s proportional cover of coniferous, broadleaf, and mixed forests, as well as grassland and shrubland, was used in a principal component analysis (PCA), to obtain two composite predictor variables of riparian plant community composition to be used in subsequent statistical analysis. Proportional cover was logit transformed before the PCA was performed, using the package ‘car’ in R (Fox & Weisberg, 2019).

**Supporting Results**

Riparian plant community composition was well distinguished in the PCA (Figure S1), with coniferous forest and grassland cover being positively associated with each other and negatively associated with cover of broadleaf forest and shrubland, which in turn were positively associated with each other, along the primary axis (PC1, explaining 32.1% of the variation), and mixed forest cover being neutral to the other plant communities and positively associated with the secondary axis (PC2, explaining 22.7% of the variation).


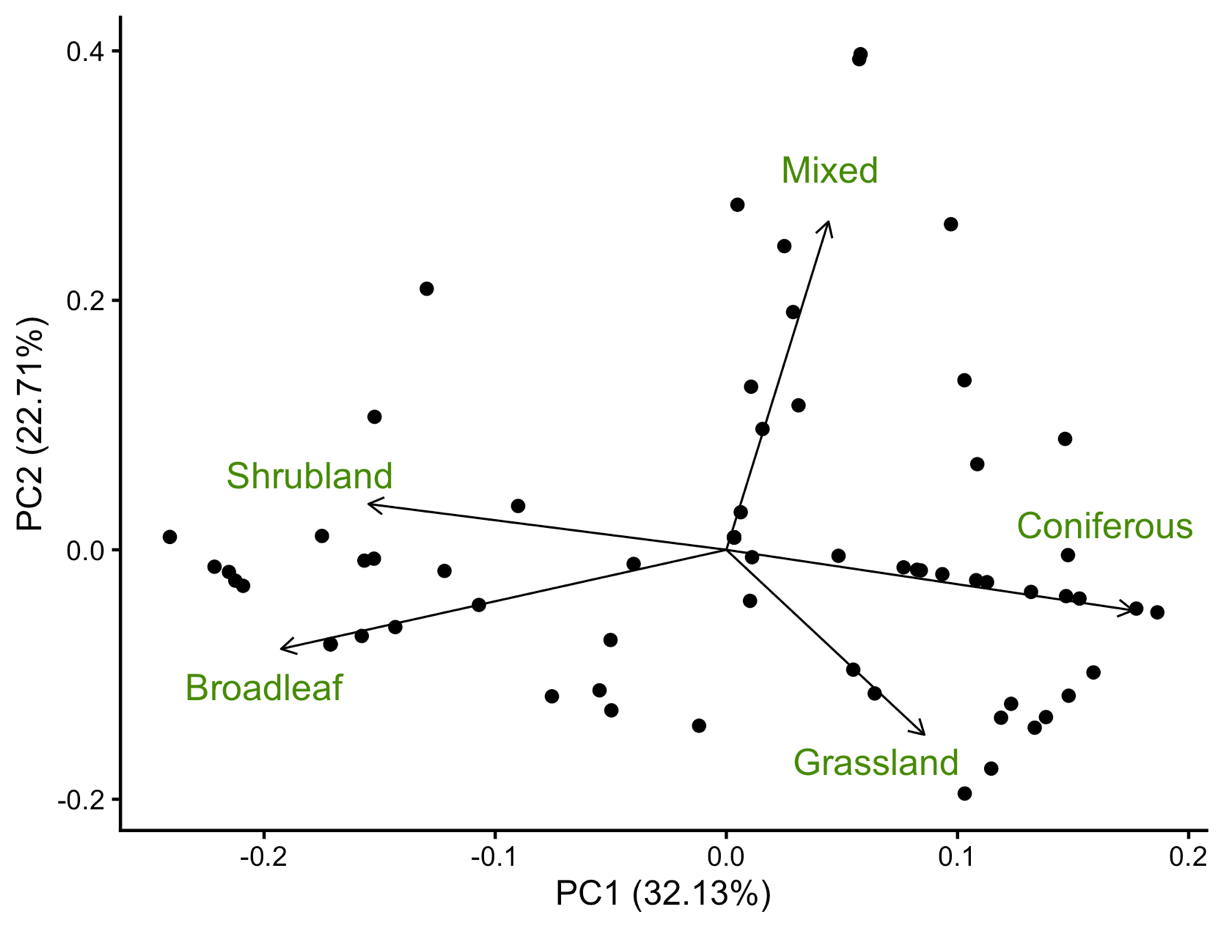


**FIGURE S1** Results from a PCA on riparian plant community composition (relative cover) across all sampling sites (black points).

There were significant differences among basins in both MAT (*χ*^2^ = 52.169, *p* < 0.001; Figure S2a) and temperature variability (*χ*^2^ = 61.146, *p* < 0.001; Figure S2b). MAP also varied significantly among basin (*χ*^2^ = 58.369, *p* < 0.001; Figure S2c) as did the variability in precipitation (*χ*^2^ = 61.146, *p* < 0.001; Figure S2d). Channel width, however, did not show a significant difference among basins (*F* = 1.474, *p* = 0.201; Figure S3a) and neither did channel depth (*F* = 1.545, *p* = 0.178) or canopy cover (*χ*^2^ = 7.083, *p* = 0.313; Figure S3b). Human population density differed significantly among basins (*χ*^2^ = 20.106, *p* = 0.003; Figure S3c), whereas LUI did not (*F* = 1.371, *p* = 0.240; Figure S3d). For mean values and pairwise comparisons among basins, see Table S6.


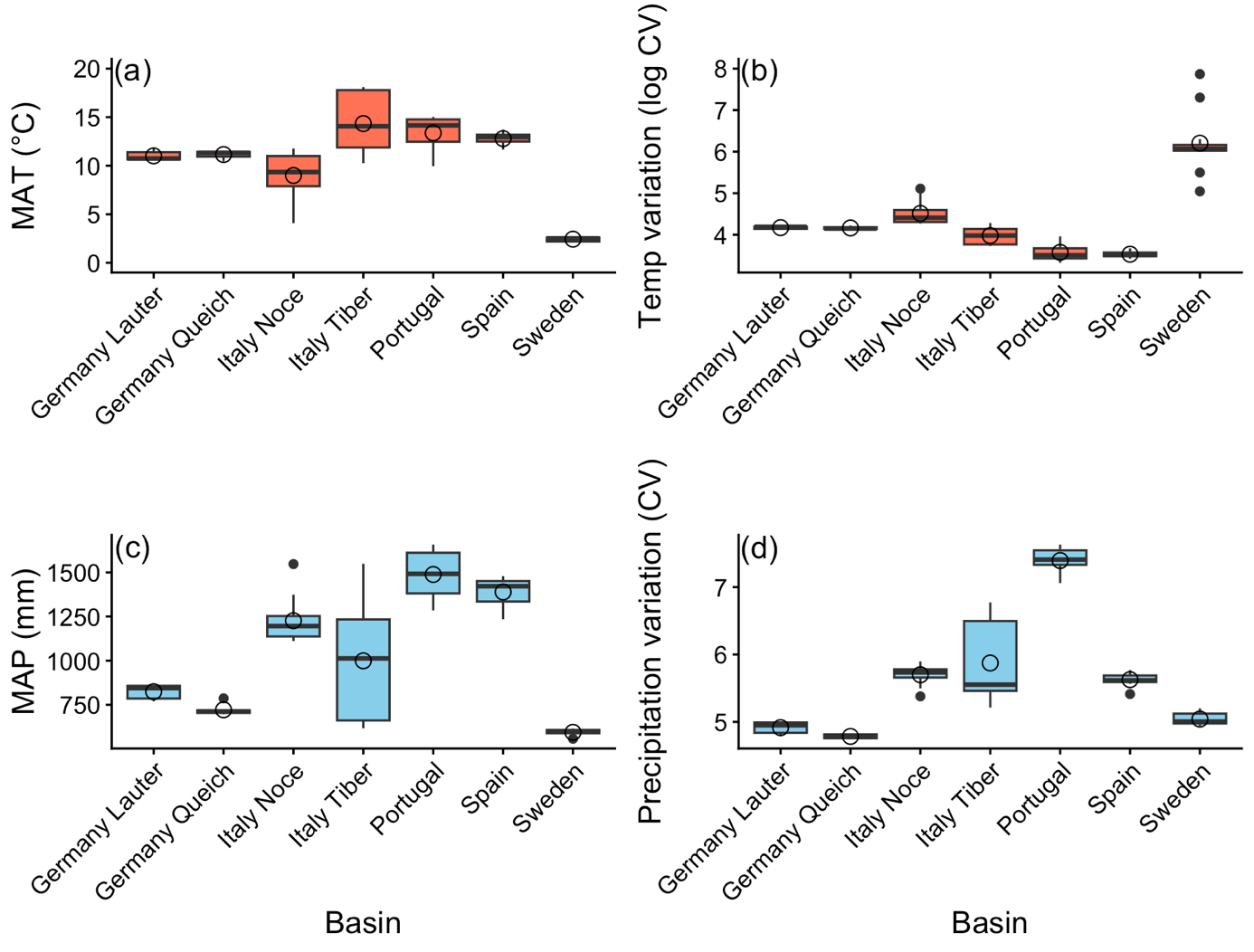


**FIGURE S2** Boxplots of (a) mean annual temperature (MAT), (b) temperature variability (CV), (c) mean annual precipitation (MAP), and (d) variability (CV) in precipitation in seven basins across Europe. The boxplots show the minimum and maximum excluding outliers, first quartile (median of the lower half), median, and third quartile (median of upper half). Open circles represent the mean value for each basin.


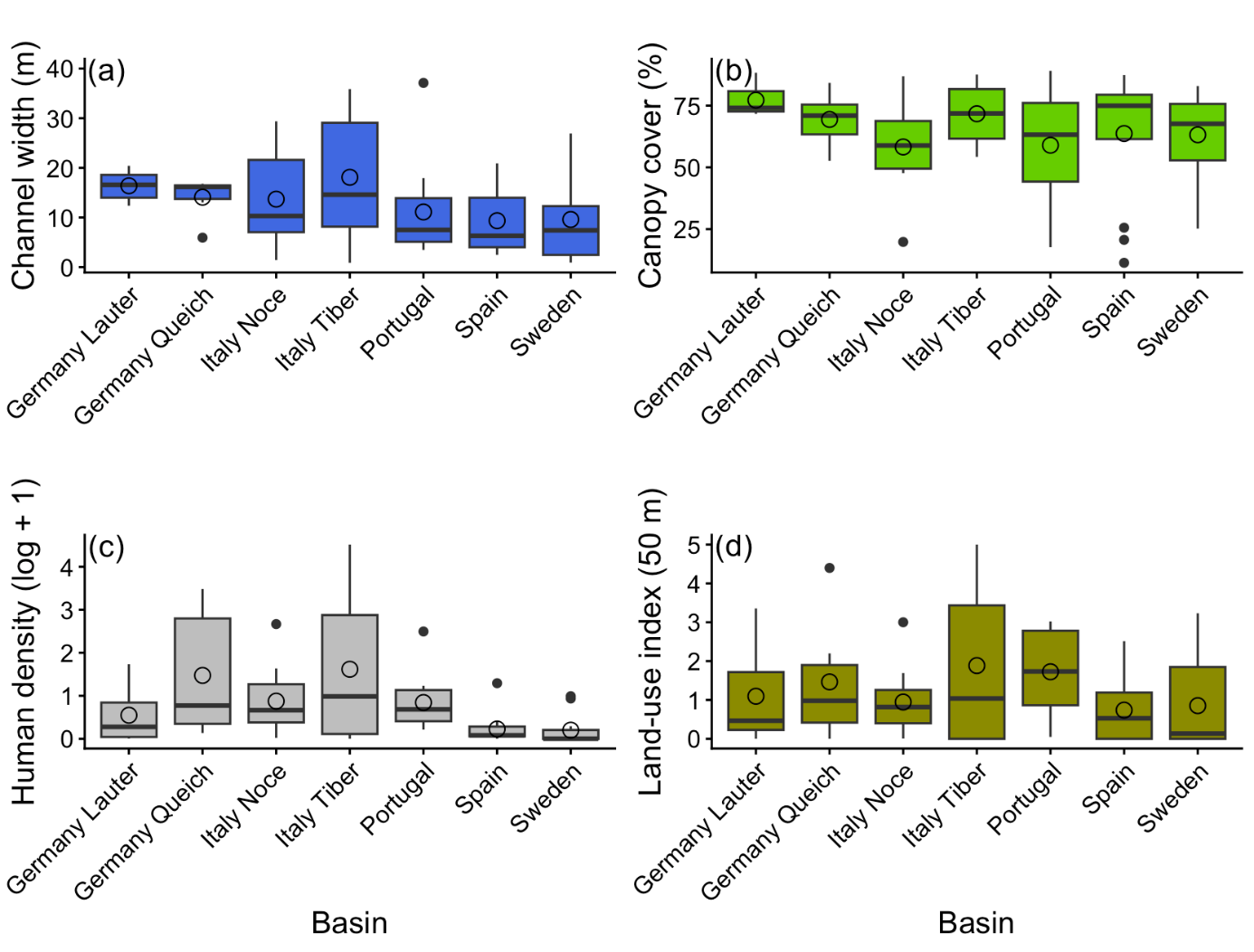


**FIGURE S3** Boxplots of (a) channel width, (b) canopy cover, (c) human population density, and (d) land-use intensity in seven basins across Europe. The boxplots show the minimum and maximum excluding outliers, first quartile (median of the lower half), median, and third quartile (median of upper half). Open circles represent the mean value for each basin.

**Supporting references**

Andersson, S., Bärring, L., Landelius, T., Samuelsson, P., & Schimanke, S. (2021). SMHI gridded climatology.

Ballabio, C., Panagos, P., & Monatanarella, L. (2016). Mapping topsoil physical properties at European scale using the LUCAS database. *Geoderma*, 261, 110–123. DOI: https://doi.org/10.1016/j.geoderma.2015.07.006

Benda, L., Miller, D., & Barquín, J. (2011). Creating a catchment scale perspective for river restoration. *Hydrology and Earth System Sciences*, 15, 2995–3015. DOI: https://doi.org/10.5194/hess-15-2995-2011

Benda, L., Miller, D., Andras, K., Bigelow, P., Reeves, G., & Michael, D. (2007). NetMap: a new tool in support of watershed science and resource management. *Forest Science*, 53, 206–219. DOI: https://doi.org/10.1093/forestscience/53.2.206

Fox, J., & Weisberg, S. (2019). *An R Companion to Applied Regression*, Third edition. Sage, Thousand Oaks CA. https://www.john-fox.ca/Companion/.

Gomes, G., Thiemig, V., Skøien, J. O., Ziese, M., Rauthe-Schöch, A., Rustemeier, E., ... & Salamon, P. (2020). *EMO: A high-resolution multivariable gridded meteorological data set for Europe* [dataset]. DOI: 10.2905/0BD84BE4-CEC8-4180-97A6-8B3ADAAC4D26

Hengl, T. (2021). *Continental Europe surface lithology based on EGDI / OneGeology map at 1:1M scale (v1.0)* [Data set]. Zenodo. DOI: https://doi.org/10.5281/zenodo.4787632

Ilhardt, B. L., Verry, E. S., & Palik, P. J., (2000). Defining riparian areas. In: Verry, E. S., Hornbeck, J. W., Dollof, C. A. (Eds), *Riparian Management in Forests of the Continental Eastern United States*. Lewis Publishers, Boca Raton, FL, USA.

Isenburg, M. (2008). *LASTOOS: LasTools-Efficient Tools for LiDAR Processing*, Version 111216, http://lastools.org. (Consulted on the 12 of December of 2023).

Lang, N., Jetz, W., Schindler, K., & Wegner, J. D. (2023). A high-resolution canopy height model of the Earth. *Nature Ecology & Evolution*, 7, 1778-1789. DOI: https://doi.org/10.1038/s41559-023-02206-6

Raffa, M., Reder, A., & Mercogliano, P. (2021). *D4. 1 Datasets of downscaled ERA5 reanalysis over Italy*. Technical Report of the Highlander project.

Rosgen, D. L., (1996). Applied river morphology. *Wildland Hydrology*. Pagosa Springs, CO, USA.
